# Effects of Somatic Methylation in Colonic Polyps on Risk of Developing Metachronous Advanced Colorectal Lesions

**DOI:** 10.3390/cancers13020246

**Published:** 2021-01-11

**Authors:** Oscar Murcia, Alejandro Martínez-Roca, Miriam Juárez, Mar Giner-Calabuig, Miren Alustiza, Cristina Mira, Carolina Mangas-Sanjuan, Eva Serrano, Francisco Antonio Ruiz-Gómez, Sandra Baile-Maxia, Lucía Medina, Cristina Alenda, Artemio Payá, María Rodriguez-Soler, Pedro Zapater, Rodrigo Jover

**Affiliations:** 1 Instituto de Investigación Sanitaria ISABIAL, Servicio de Medicina Digestiva, Hospital General Universitario de Alicante, 03010 Alicante, Spain; cristina14.eii@gmail.com (C.M.); cmangassanjuan@gmail.com (C.M.-S.); evaserranodiaz@hotmail.es (E.S.); ruiz.f7@gmail.com (F.A.R.-G.); sandrabm_92@hotmail.com (S.B.-M.); lucia.medinaprado@gmail.com (L.M.); nalongui@hotmail.com (M.R.-S.); 2 Unidad de Investigación, Instituto de Investigación Sanitaria ISABIAL, Hospital General Universitario de Alicante, 03010 Alicante, Spain; alex27693@gmail.com (A.M.-R.); mirjuaque@gmail.com (M.J.); mar.giner-calabuig@yale.edu (M.G.-C.); mirenalustiza92@gmail.com (M.A.); 3 Section of Digestive Diseases, Yale University, New Haven, CT 06520, USA; 4 Instituto de Investigación Sanitaria ISABIAL, Department of Pathology, Hospital General Universitario de Alicante, 03010 Alicante, Spain; alenda.cris@gmail.com (C.A.); paya_art@gva.es (A.P.); 5 Instituto de Investigación Sanitaria ISABIAL, Department of Clinical Pharmacology, Hospital General Universitario de Alicante, 03010 Alicante, Spain; pzapater@goumh.umh.es

**Keywords:** methylation, metachronous advanced colorectal lesions, polyps, CIMP

## Abstract

**Simple Summary:**

Post-polypectomy endoscopic surveillance is predominantly based on the size and number of polyps found at baseline. The utility of molecular markers for predicting the risk of metachronous advanced lesions remains poorly investigated. Patients with CpG island methylator phenotype (CIMP)+ polyps show a higher risk of developing advanced lesions at follow-up. This fact is independent of polyp size and other factors classically related to advanced lesion development. Addition of CIMP status improved the metachronous advanced colorectal lesion (MACL) risk estimation, especially the sensitivity. CIMP may be a useful marker for endoscopic surveillance after polypectomy.

**Abstract:**

The utility of molecular markers for predicting the risk of metachronous advanced colorectal lesions (MACLs) remains poorly investigated. We examined the relationship between somatic hypermethylation in polyps at baseline and the risk of developing MACL. This retrospective cohort study included 281 consecutive patients with colonic polyps who were enrolled between 2007 and 2009 and followed-up until 2014. MACLs were defined as adenomas of ≥10 mm, high-grade dysplasia, or with a villous component; and serrated lesions of ≥10 mm or with dysplasia. In total, 595 polyps were removed at baseline colonoscopy and analyzed for pathological characteristics and CpG island methylator phenotype (CIMP) using the MS-MLPA (Methylation-Specific -- Multiplex Ligation-dependent Probe Amplification) technique. Forty-five patients (16.0%) showed at least one CIMP+ polyp. MACL risk was higher in patients with CIMP+ polyps (odds ratio (OR), 4.50; 95% CI, 1.78–11.4; *p* = 0.002). Patients with CIMP+ polyps also exhibited shorter time to MACL development (33.8 months vs. 50.1 months; *p* < 0.001), even with adjustment for polyp size and number (OR, 2.40; 95% CI, 1.33–4.34). Adding CIMP analysis improved the sensitivity (57.0% to 70.9%), negative predictive value (71.1% to 77.3%), and overall accuracy (49.8% to 52.0%) for MACL risk estimation. These results highlight that CIMP may be a useful marker for endoscopic surveillance.

## 1. Introduction

Colorectal cancer (CRC) is a major cause of cancer morbidity and death in developed countries. Its incidence and mortality can be reduced by endoscopic removal of CRC precursor lesions, adenomas, or serrated polyps [1,2]. However, patients who undergo removal of advanced adenomas or serrated polyps during colonoscopy carry an increased risk of developing metachronous lesions and, eventually, CRC. Thus, surveillance colonoscopy is recommended for patients who present with advanced polyps at baseline [3,4].

In the guidelines for surveillance after polyp excision, patients are stratified into risk groups mainly according to characteristics of the lesions found at the index colonoscopy [4]. The rationale behind such categorization is that the advanced neoplasia risk at follow-up colonoscopy depends on the number, size, and histology of polyps at baseline. However, other polyp characteristics may also be related to the risk of developing advanced lesions and could be useful for identifying high-risk patients. To date, few studies have investigated the relationship between molecular markers and risk of developing metachronous neoplasia at follow-up. Some prior studies demonstrate that somatic mutation in *KRAS* (**K**irsten **RA**t **S**arcoma virus) gene may predict metachronous advanced colorectal lesions (MACLs) during colonoscopic surveillance [5]. Additionally, studies applying the consensus molecular subtypes (CMSs) of CRC to polyps have indicated that certain genetic anomalies may be markers of future CRC risk [6,7].

Recent evidence has highlighted that high-level methylation of CpG islands in certain DNA regions is important in CRC oncogenesis [8], but its role as a prognostic marker remains controversial [9,10]. Until now, it has not been investigated whether aberrant methylation in polyps is a molecular marker of risk of metachronous neoplasia. In the present study, we aimed to evaluate whether the presence of CIMP+ polyps in adenomatous and serrated lesions at baseline colonoscopy could predict MACL risk at follow-up.

## 2. Results

### 2.1. Characteristics of the Study Population

**Figure 1:**
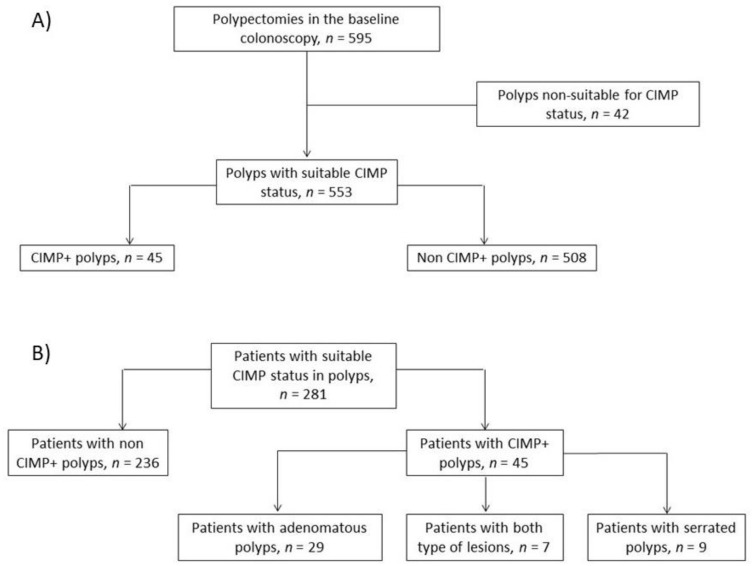
Study flow charts. (**A**) Flow chart of polyps removed at baseline colonoscopy. (**B**) Flow chart of the study population according to the genetic profile of polyps at baseline.

Among the 281 patients, 177 (63.0%) were men, and the median age at recruitment was 65 years (range, 28–90 years). The median follow-up duration was 36 months (interquartile range (IQR), 25–48 months), with a median of two surveillance colonoscopies (range, 1–6). At baseline, there were no patients with more than one methylated polyp. Figure 1 shows the flow chart of patient inclusion and of the CIMP status of polyps removed at baseline colonoscopy. Twenty-four patients died throughout the follow-up period. A total of 72 patients (25.6%) had previously been diagnosed with polyps, and 41 patients (14.6%) had previous CRC—19 at stage I, 10 at stage II, and 12 at stage III.

### 2.2. Characteristics of Polyps from Baseline Colonoscopy According to CIMP Status

CIMP status was investigated for 595 polyps removed at the baseline colonoscopy. A total of 42 polyps were excluded due to DNA degradation. Table 1 shows the between-group comparisons of anatomopathological and clinical features. The samples included 407 adenomatous polyps, of which 32 (7.8%) were CIMP+, and 146 serrated polyps, of which 13 (8.9%) were CIMP+.

### 2.3. Patients’ Characteristics According to CIMP Status

Patients with and without CIMP+ polyps did not differ in terms of age, sex, smoking history, history of CRC, or presence of *BRAF* (Raf murine sarcoma viral oncogene homolog B) or KRAS mutated polyps. Compared to patients without CIMP+ polyps, those with CIMP+ polyps exhibited a higher number of polyps at baseline and a higher number of colonoscopies performed (Table 2). Table 2 also shows the different reasons for repeating colonoscopy.

### 2.4. Characteristics of MACL and Risk According to CIMP Status of Polyps at Baseline Colonoscopy

A total of 86 patients developed MACL during follow-up. Of them, 49 patients developed an adenomatous advanced lesion, whereas advanced serrated lesions were found in 34 patients. Moreover, three patients developed CRC throughout the surveillance, two of them being a recurrence (both at stage I).

Patients with CIMP+ polyps showed a higher proportion of MACL throughout surveillance (30.2% vs 9.7%; *p <* 0.001, Table 2). Table 3 presents other factors related to MACL development. In the univariate analysis, higher risk of MACL was associated with smoking history, history of CRC, number of polyps, KRAS+ status, and CIMP+ status. However, in the multivariate analysis, logistic regression showed that CIMP+ was the only independent factor associated with MACL development (odds ratio (OR), 4.50; 95% CI, 1.78–11.4) (Table 3). Similar results were obtained when we separately analyzed patients with only adenomatous or only serrated polyps (Table S1). In the multivariate analysis, CIMP+ was associated with higher MACL risk in patients with adenomatous polyps (OR, 2.31; 95% CI, 1.00–5.36; *p* = 0.049) as well as in patients with serrated lesions (OR, 10.3; 95% CI, 1.05–102.2; *p* = 0.046).

**Figure 2:**
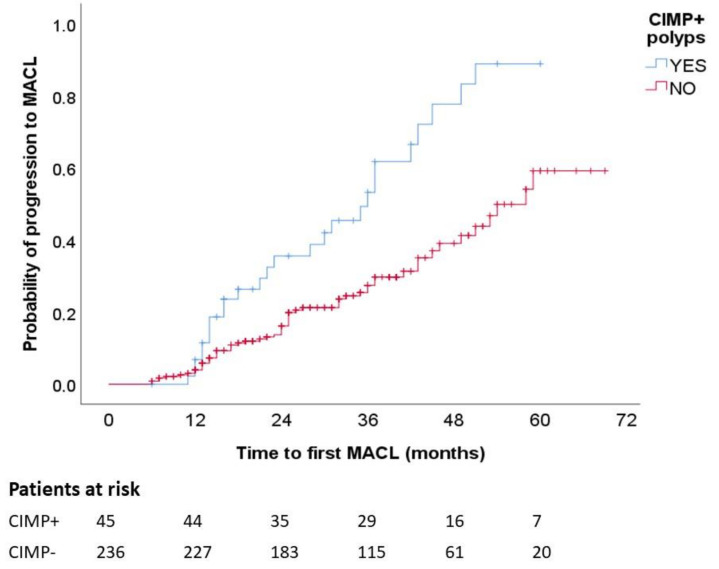
MACL risk according to CIMP status. Kaplan–Meier survival curves illustrate higher proportion of MACL among patients with CIMP+ polyps. MACL, metachronous advanced colorectal lesion. *p* < 0.001.

Figure 2 presents the Kaplan–Meier chart showing that patients with CIMP+ polyps had a significantly higher frequency of MACL at follow-up (*p* log-rank, <0.001). In the multivariate analysis with adjustment for number of colonoscopies, shorter time until MACL diagnosis was independently associated with CIMP+ (hazard ratio (HR), 2.40; 95% CI, 1.33–4.34) and male sex (HR, 1.99; 95% CI, 1.04–3.78) (Table 4).

A separate analysis of patients with adenomatous and serrated polyps also revealed shorter intervals to MACL diagnosis in patients with CIMP+ adenomas (*p* log-rank, 0.009) (Figure S1A) and in patients with CIMP+ serrated polyps (*p* log-rank, 0.042) (Figure S1B). In the multivariate analysis including patients with adenomatous polyps, higher risk of MACL development was associated with CIMP+ status (HR, 2.78; 95% CI, 1.19–6.47) and the presence of three or more polyps (HR, 3.83; 95% CI, 1.81–8.13) (*p* < 0.001 for both) (Table S2).

### 2.5. Usefulness of CIMP Assessment for MACL Risk Estimation

We added CIMP analysis to the classical risk factors used to estimate the risk of MACL development, including size of ≥10 mm, number ≥3, high-grade dysplasia, or villous component for adenomas; or size of ≥10 mm or presence of dysplasia for serrated polyps. The addition of CIMP status improved the performance of classical risk factors for the estimation of MACL risk at follow-up, increasing sensitivity from 57.0% to 70.9%, negative predictive value from 71.1% to 77.3%, and overall accuracy from 49.8% to 52.0% (Table 5).

## 3. Discussion

The results of our study suggest that the CIMP status of polyps removed at baseline may be a predictor of future MACL development during follow-up. This predictive value was independent of polyp size and other factors classically related to advanced lesion development and was demonstrated for both adenomatous and serrated polyps. Addition of CIMP status also improved the MACL risk estimation compared to the classical risk factors alone, especially the sensitivity. Overall, our findings establish the potential utility of this molecular marker for stratifying risk among patients with colonic polyps and, thus, optimizing their surveillance.

Previous studies have investigated the role of molecular markers in polyps for predicting the development of metachronous lesions, especially advanced adenomas, revealing an increased MACL risk in patients with KRAS-mutated polyps at baseline [5]. Concerning serrated lesions, a recent study has found no association between BRAF mutations or CIMP status in polyps at baseline and metachronous advanced lesions [11]. Similar results were found regarding BRAF by Juárez et al. [11]. However, our study highlights different results for CIMP. We have now found that CIMP status can also predict MACL development in our population. However, this discrepancy can be partially explained by the different characteristics of the patients included in both studies, with the study of Hua including only patients with serrated lesions, the majority of them with only hyperplastic polyps, and also with a low rate of patients with CIMP+ lesions [11].

It is plausible that molecular markers could predict MACL risk since these markers may identify polyps having features related to the main causes of metachronous neoplasia development following colonic polyp removal. Such causative factors may include missed or incompletely resected lesions in low-quality baseline procedures or fast-growing biological characteristics of the lesions, which could promote rapid growth and progression to advanced states. Both of these conditions could be potentially related to CIMP+ lesions. On one hand, CIMP+ lesions are traditionally related to flat or laterally spread right-sided polyps [12,13,14], with lesions that are commonly difficult to detect and resect—factors that are strongly linked to colonoscopy quality. On the other hand, CIMP+ lesions have been associated with fast oncogenic growth, especially when coexisting with other molecular alterations such as microsatellite instability [15,16].

In our study, CIMP+ lesions were found among both adenomatous and serrated polyps, suggesting that CIMP+ status was not limited to the serrated pathway of carcinogenesis. Previous studies have reported that adenomas classically show low methylation levels, or even no methylation [17]. However, this is controversial as other series show proportions of CIMP+ adenomatous ranging from 10% to 30% of samples [18,19,20,21,22,23,24], similar to our present findings. There are several possible explanations for this discrepancy. One possible reason is the use of different techniques and criteria for classifying lesions as CIMP+ [25,26,27,28]. The classical approach by quantitative real-time polymerase chain reaction (Methy-Light) evaluates hypermethylation in a five-marker panel (*CACNA1G*, *CDKN2A* (p16), *CRABP1*, *MLH1*, and *NEUROG1*) [28,29]. However, the MS-MLPA technique has already been validated as a reliable and cost-effective method for CIMP assessing in CRC in several studies, with similar overall accuracy [30,31,32,33]. Moreover, MS-MLPA analyzes the same five markers as Methy-Light, adding *IGF2, SOSC1*, and *RUNX3* for CIMP analysis. On the other hand, it is also possible that there is some degree of heterogeneity in the characteristics of the analyzed samples [34]. Notably, other studies using the same definition of CIMP+ adenomas reported results similar to ours, finding 17% CIMP+ adenomas [24].

We analyzed additional aspects—including size, high-grade dysplasia, and villous component—and observed no association between these characteristics and MACL development in our retrospective cohort. Only multiplicity (presence of ≥3 polyps) was associated with higher proportions of MACL (*p* = 0.001) in the univariate analysis. Notably, CIMP remained a marker of MACL risk in adenomatous and serrated polyps, independently of these other features. Moreover, CIMP status was not related to size or other advanced features, indicating that assessment of this molecular marker extends beyond traditional indicators of risk, adding potentially useful complementary information. We demonstrated that the addition of CIMP status led to improved sensitivity for MACL risk estimation compared to with classical risk factors alone, suggesting that analysis of this molecular marker may improve the selection of patients who require colonoscopy surveillance due to their higher risk of developing MACL. There remains a need for cost effectiveness studies of this issue. However, the cost of molecular analysis of polyps is quickly decreasing with the development of next-generation sequencing technology, making this option feasible if our results are confirmed.

Our study has some limitations. As it is a retrospective observational study, it could show some biases inherent to this kind of study. There is the possibility of selection bias; since the data were not prospectively collected, we could have selected patients with higher risk of MACL development, thus increasing the potential role of any new marker investigated. There is also the possibility of information bias, including the lack of some data that could also be related to advanced lesion development. For instance, information regarding smoking status and body mass index were not adequately recorded for the study participants. Additionally, our study was not limited to surveillance. A substantial proportion of our patients underwent repeated colonoscopy due to symptoms or fecal immunochemical test (FIT)-based CRC screening, increasing the heterogeneity of our sample, making it difficult to apply our results to the specific scenario of surveillance after polyp excision. Other limitations include the small sample size in some subgroups, particularly in patients with serrated lesions and CIMP+ polyps, as well as patients with CRC at follow-up, which prevented more robust results. Finally, we did not identify any association between MACL and several classical risk factors such as polyp size. This could also be related to the retrospective characteristics of the study and the limited sample size.

## 4. Materials and Methods

### 4.1. Study Design and Population

This single-center retrospective cohort study included a total of 281 patients. We retrospectively recruited patients who were diagnosed with polyps during a colonoscopic examination at the Hospital General Universitario of Alicante, Spain, between 2007 and 2009. All included patients had undergone at least one surveillance colonoscopy more than 6 months after the baseline examination. Repeated colonoscopies were mainly performed due to the presence of symptoms, after fecal immunochemical test (FIT)-based CRC screening, or as surveillance after excision of CRC or adenomas. Data from surveillance colonoscopies were collected until December 2014. We also collected the patients’ clinicopathological information and personal history. Exclusion criteria were: CRC diagnosis at study inclusion; previous diagnosis of polyposis syndrome, Lynch syndrome, or inflammatory bowel disease; a score of <2 on the Boston Bowel Preparation Scale at any colonic segment; or DNA degradation of polyps verified by the NanoDrop system (Thermo Fisher Scientific, Waltham, MA, USA). All patients’ clinical data were anonymized, and this study was approved by the Ethics Committee of the Hospital General Universitario of Alicante (ID PI2015/27), Spain. All authors had access to the study data and reviewed and approved the final manuscript.

### 4.2. Samples

A total of 595 polyps were endoscopically removed during the baseline colonoscopies of the 281 patients. From paraffin-embedded polyp tissue, samples were prepared by microdissection into ten 5-μm thick sections. For each polyp, we collected data about its genetic profile and histopathological aspects, including size, number, morphology, pathology, and location. Samples were reviewed by two independent expert pathologists focused on gastrointestinal oncology (C.A. and A.P.). Polyps removed from the cecum, right colon, or transverse colon were considered proximal polyps, while those removed from the rectum, sigmoid colon, or left colon, including the splenic flexure, were considered distal polyps.

At follow-up colonoscopies, we considered as MACL any CRC, advanced adenoma, or advanced serrated lesion. Advanced adenomas included those with a size of ≥10 mm, high grade of dysplasia, or villous component. Advanced serrated lesions were defined by a size of ≥10 mm or lesions with any grade of dysplasia [35].

### 4.3. DNA Extraction

DNA was extracted from formalin-fixed paraffin-embedded (FFPE) samples using the QIAamp DNA Investigator kit (QIAGEN, Hilden, Germany) and the EZNA Forensic DNA kit (OMEGA Bio-tek, Norcross, GA, USA), following the manufacturers’ protocols.

### 4.4. CIMP Analysis

We assessed the level of aberrant methylation using the MS-MLPA technique and the SALSA MLPA Probemix ME042-CI CIMP kit (MRC Holland). The PCR reaction contained DNA, HotStar Taq polymerase, forward primers, biotinylated reverse primers, and water. Using MS-MLPA technology, we analyzed eight markers: *CACNA1G, CDKN2A (p16), CRABP1, IGF2, MLH1, NEUROG1, RUNX3,* and *SOCS1* [36]. We set a cutoff of 20% of probes for each marker and a ratio of 0.25 between case and control samples for each probe to be considered positively methylated. For *CACNA1G, IGF2*, and *RUNX3* genes, three probes were used to test for methylation. For *CDKN2A (p16), CRABP, MLH1*, and *SOCS1* genes, four probes, and, finally, for *NEUROG1,* six probes were used (Table S3). All these data were analyzed using GeneMapper software v6.0 (Thermo Fisher Scientific, Waltham, MA, USA). A polyp showing six or more methylated markers was considered CIMP+, while a polyp showing methylation of 5 or less markers was considered as CIMP−. We stablished a cutoff of 25% of polyps with hypermethylation at baseline colonoscopy for considering a patient as CIMP+.

### 4.5. Other Genetic Analysis

To assess the potential contribution of polyp CIMP status to MACL development risk, we also examined polyps for mutations in the BRAF and KRAS genes. We identified *BRAF* mutations at codon 600 (V600E) using real-time PCR (ABI PRISM 7500; Applied Biosystems, Foster City, CA, USA) based on the allelic discrimination method (Applied Biosystems, Foster City, CA, USA) and using specific TaqMan probes [37]. We identified *KRAS* mutations at exon 2 (including codons 12 and 13) by performing DNA Sanger sequencing (ABI3500 Genetic Analyzer; Applied Biosystems), as previously described [37]. Patients were considered BRAF-mut or KRAS-mut if they had at least one polyp showing a somatic BRAF or KRAS mutation, respectively.

### 4.6. Statistical Analysis

All data were analyzed using SPSS 25.0 software (IBM, Chicago, IL, USA). In the descriptive analyses, parametric continuous variables were reported as mean ± standard deviation (SD), and non-parametric continuous variables as median and interquartile range (IQR). Categorical variables were expressed as absolute frequency and percentage.

For comparative analyses, patients were classified according to their CIMP status at baseline colonoscopy. Additional analyses compared patients with adenomas or serrated lesions; these analyses excluded patients having both types of lesions. The chi-square test was used to compare these groups according to sex, presence of genetic alterations in the polyps, previous CRC, previous smoking, and other baseline categorical features. Variables with more than three categories were analyzed using an ANOVA test. Baseline age, polyp size, polyp number, and other quantitative variables were compared using the Student’s t-test, after applying Levene’s test for parametric variables, or the Mann–Whitney U test for non-parametric variables. We also used the same methods to compare polyps from the baseline colonoscopy in terms of several variables, according to CIMP status.

For both groups of patients, we analyzed MACL risk according to CIMP status using univariate logistic regression. We additionally analyzed other variables such as age, sex, previous smoking, history of CRC, mutations in KRAS and BRAF, and classical MACL risk factors (>3 polyps, villous component, and lesion size) and included these variables in the multivariate analysis if *p* was <0.20 [38]. The multivariate analysis was performed using a binary logistic regression model, showing the results as odds ratio (OR) and 95% confidence interval (CI).

We analyzed MACL development over time according to the presence of aberrant methylation in polyps using Kaplan–Meier survival curves and the log-rank test. Patients were censored after developing an MACL, whereas the patients who died during the follow-up period were excluded from this analysis. Again, a multivariate analysis was carried out using a Cox regression model with adjustment for variables with *p* values of <0.20. These results are shown as median of time (months), hazard ratio (HR), and 95% CI. All *p* values are two-sided, and a *p* value of <0.05 was considered statistically significant.

## 5. Conclusions

In summary, our present results suggest that analysis of the CIMP status of polyps removed at baseline colonoscopy could be a useful marker for assessing MACL risk. A CIMP+ pattern in polyps (both adenomatous and serrated lesions) at the baseline exploration was associated with higher risk of MACL development. Future prospective cohort studies with a larger sample size are needed to confirm and validate our findings. If our results are confirmed, analysis of the genetic profile of polyps could add useful information that may help to adequately ascertain a patient’s risk of developing advanced lesions at follow-up, thus influencing surveillance recommendations.

## Figures and Tables

**Table 1:** Characteristics of polyps at baseline colonoscopy according to CpG island methylator phenotype (CIMP) status.

Variable	CIMP+ Polyps, *n* = 45	CIMP− Polyps, *n* = 508	*p* Value
Adenomatous lesions, *n* (%)	32 (71.1)	375 (73.8)	0.189
Tubular adenomas, *n* (%)	24 (53.3)	327 (64.4)	
Villous adenomas, *n* (%)	0 (0)	2 (0.4)	
Tubulovillous adenomas, *n* (%)	8 (17.8)	46 (9.1)	
High grade of dysplasia, *n* (%)	5 (11.1)	30 (5.9)	0.276
Serrated lesions, *n* (%)	13 (28.9)	133 (26.2)	0.634
Hyperplastic lesions, *n* (%)	10 (22.2)	106 (20.9)	
Sessile serrated lesions, *n* (%)	3 (6.2)	19 (3.7)	
Traditional serrated lesions, *n* (%)	0 (0)	8 (1.6)	
Right location, *n* (%)	8 (17.9)	160 (31.5)	0.078
Size, *n* (%)	--	--	0.106
<5 mm, *n* (%)	1 (2.6)	70 (13.8)	
5–9 mm, *n* (%)	19 (42.1)	218 (42.9)	
>9 mm, *n* (%)	25 (55.6)	220 (43.4)	
Advanced adenomatous lesions, *n* (%)	26 (57.8)	253 (49.8)	0.364
Advanced serrated lesions, *n* (%)	14 (31.1)	135 (26.6)	0.799

**Table 2:** Patients’ characteristics according to CIMP status of polyps at baseline colonoscopy.

Variables	All Patients (*n* = 281)	Patients with CIMP+Polyps (*n* = 45)	Patients without CIMP+Polyps (*n* = 236)	*p* Value
Age, mean (SD)	65.2 (11.9)	65.3 (11.9)	65.2 (11.9)	0.976
Male sex, *n* (%)	177 (63.0)	27 (60.0)	150 (63.6)	0.650
Number of polyps at baseline, mean (SD)	2.9 (2.6)	3.9 (3.6)	2.7 (2.4)	0.004
Number of colonoscopies, mean (SD)	1.9 (1.1)	2.4 (1.4)	1.8 (1.0)	0.007
Indication for repeat colonoscopy, *n* (%)	--	--	--	--
FIT positive	66 (23.5)	10 (22.2)	56 (23.7)	0.469
Lower GI bleeding	62 (22.1)	11 (24.4)	51 (21.6)	0.786
Post-polypectomy surveillance	58 (20.6)	5 (11.1)	53 (22.5)	0.058
Colorectal cancer surveillance	41 (14.6)	14 (31.1)	27 (11.4)	0.007
Chronic diarrhea	12 (4.3)	1 (2.2)	11 (4.7)	0.680
Other	42 (14.9)	4 (8.9)	38 (16.1)	0.817
Previous smokers, *n* (%)	62 (22.1)	15 (33.3)	47 (19.9)	0.114
Previous CRC, *n* (%)	41 (14.6)	7 (15.6)	34 (14.4)	0.713
BRAF-mut polyps, *n* (%)	44 (15.7)	11 (24.4)	33 (14.0)	0.207
KRAS-mut polyps, *n* (%)	72 (25.6)	16 (35.6)	56 (23.7)	0.248
Advanced polyps at baseline, *n* (%)	153 (54.4)	27 (60.0)	126 (53.4)	0.415
Metachronous advanced colorectal lesions, *n* (%)	86 (30.6)	26 (57.8)	60 (25.4)	<0.001

SD, standard deviation; CRC, colorectal cancer; MACL, metachronous advanced colorectal lesion; FIT, fecal immunochemical test.

**Table 3:** Risk factors for metachronous advanced colorectal lesion (MACL).

	Metachronous Advanced Colorectal Lesion					
	Univariate Analysis			Multivariate Analysis		
Variable	MACL, *n* = 86 (30.6%)	No MACL, *n* = 195 (69.4%)	*p* Value	OR	95% CI	*p* Value
Age, mean (SD)	65.8 (11.5)	65.0 (12.0)	0.640			
Male sex, *n* (%)	58 (67.4)	119 (61.0)	0.305			
Previous smokers, *n* (%)	60 (21.4)	23 (8.2)	0.020	2.81	0.94–8.37	0.064
Previous CRC, *n* (%)	18 (20.9)	23 (11.8)	0.048	1.48	0.55–3.99	0.435
BRAF-mut polyps, *n* (%)	13 (15.1)	31 (15.9)	0.636			
KRAS-mut polyps, *n* (%)	31 (36.0)	41 (21.0)	0.002	2.22	0.96–5.15	0.063
CIMP+ polyps, *n* (%)	26 (30.2)	19 (9.7)	<0.001	4.50	1.78–11.4	0.002
Polyp ≥10 mm, *n* (%)	48 (55.8)	103 (53.1)	0.674			
≥3 polyps, *n* (%)	47 (54.7)	66 (33.8)	0.001	1.87	0.87–4.04	0.112
Villous component, *n* (%)	8 (9.3)	16 (8.2)	0.762			
Advanced baseline polyps, *n* (%)	49 (57.0)	104 (53.3)	0.572			

Univariate and multivariate analysis of MACL risk according to baseline colonoscopy findings. OR, odds ratio; CI, confidence interval; MACL, metachronous advanced colorectal lesions; SD, standard deviation; CRC, colorectal cancer.

**Table 4:** Time until MACL development according to potential risk factors.

	Time until MACL Development					
	Univariate Analysis			Multivariate Analysis		
Variable		Time in Months, Mean (SD)	*p* Value	HR	95% CI	*p* Value
Sex	Male Female	45.2 (2.3) 49.3 (2.4)	0.072	1.99	1.04–3.78	0.037
Previous smoker	Yes No	27.8 (4.3) 45.1 (2.2)	0.001	1.72	0.84–3.52	0.142
Previous CRC	Yes No	44.5 (4.1) 44.2 (1.4)	0.435			
BRAF-mut polyps	Yes No	41.4 (4.1) 47.7 (1.8)	0.351			
KRAS-mut polyps, *n* (%)	Yes No	42.9 (3.0) 48.9 (2.0)	0.058	1.52	0.83–2.78	0.175
CIMP+ polyps, *n* (%)	Yes No	33.8 (2.7) 50.1 (1.9)	<0.001	2.40	1.33–4.34	0.004
Polyp ≥10 mm, *n* (%)	Yes No	46.6 (2.3) 44.8 (2.0)	0.841			
≥ 3 polyps, *n* (%)	Yes No	40.4 (2.8) 50.8 (2.0)	<0.001	1.83	0.98–3.43	0.059
Villous component, *n* (%)	Yes No	39.6 (5.1) 47.3 (1.8)	0.357			
Advanced baseline polyps, *n* (%)	Yes No	46.5 (2.3) 45.0 (2.0)	0.740			

Univariate and multivariate analysis of time until MACL development according to findings in baseline colonoscopy, with adjustment for the number of colonoscopies performed (HR, 1.22; 95% CI, 0.9–1.6; *p* = 0.200). HR, hazard ratio; CI, confidence interval; MACL, metachronous advanced colorectal lesion; SD, standard deviation; CRC, colorectal cancer.

**Table 5:** Performance characteristics of CIMP status, classical risk factors, or both combined for estimation of MACL risk after colonic polyp removal.

	Sensitivity	Specificity	PPV	NPV	Overall Accuracy
CIMP status	30.2	90.2	57.8	74.6	71.9
Classical risk factors	57.0	46.7	32.0	71.1	49.8
CIMP status + classical risk factors	70.9	43.6	35.7	77.3	52.0

PPV, positive predictive value; NPV, negative predictive value; MACL, metachronous advanced colorectal lesion.

## Supplementary Materials

The following are available online at https://www.mdpi.com/2072-6694/13/2/246/s1, Figure S1: Risk of MACL throughout surveillance according to CIMP status in patients with adenomas (A) or serrated lesions (B); Table S1: Risk for Metachronous Advanced Colorectal Lesions in Patients with Adenomatous and Serrated Polyps, Table S2: Time Until AML Diagnosis in Patients with CIMP+ Adenomatous and Serrated Lesions, adjusted for the Number of Colonoscopies Performed, Table S3: Genes and Ligation Sites for Probes for CIMP Analysis.
