# Supplementary material for: Effects of Somatic Methylation in Colonic Polyps on Risk of Developing Metachronous Advanced Colorectal Lesions
Source: Cancers (Basel). 2021 Jan 11;13(2):246. doi: 10.3390/cancers13020246 (PMC7827613; doi:10.3390/cancers13020246)
Supplement: Supplementary file 1 [file cancers-13-00246-s001.pdf]

Supplementary Materials

A)

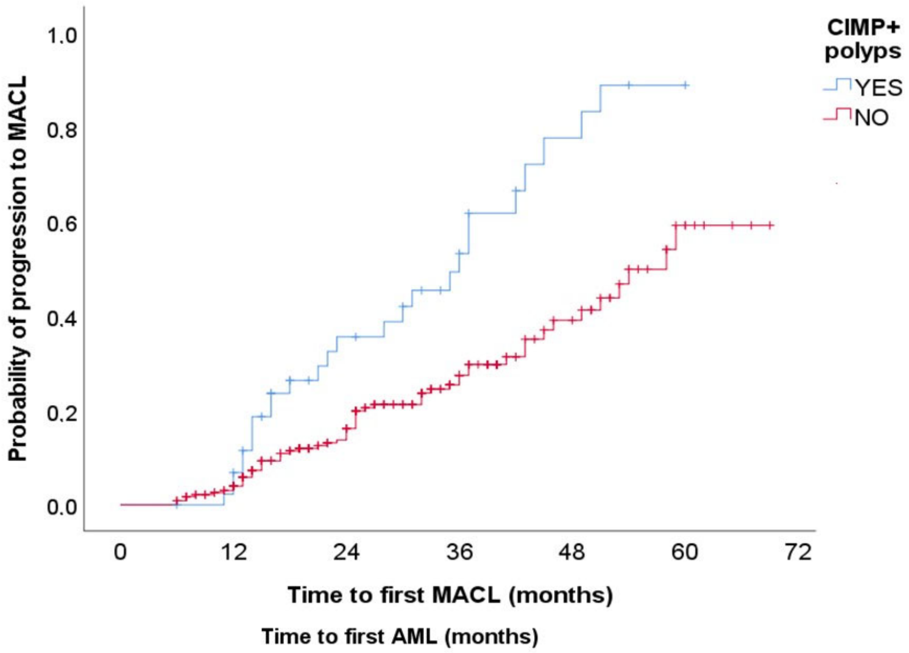

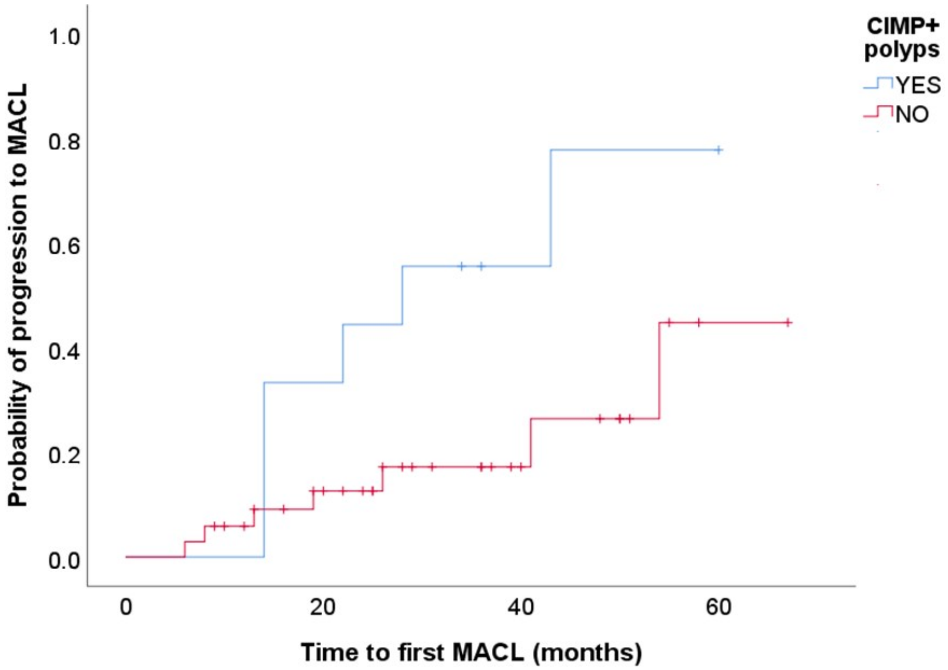

B)

Figure S1. Risk of MACL throughout surveillance according to CIMP status in patients with adenomas (*A*) or serrated lesions (*B*). Kaplan-Meier survival curves illustrate higher

proportion of MACL in patients with CIMP+ polyps. MACL, Metachronous Advanced Colorectal Lesions. $^{*}p = .009$; $^{**}p = .042$.

Table S1. Risk for Metachronous Advanced Colorectal Lesions in Patients with Adenomatous and Serrated Polyps.

**Patients with adenomatous polyps at baseline colonoscopy**						
	**Univariate analysis**			**Multivariate analysis**		
Variable	**Yes, n = 58 (30.5%)**	**No, *n* = 132 (69.5%)**	***p* value**	**OR**	**95% CI**	***p* value**
Age, mean (SD)	64.5 (10.4)	67.3 (12.0)	.227			
Male sex, *n* (%)	38 (65.5)	84 (63.6)	.803			
Previous smokers, *n* (%)	4 (12.5)	5 (8.2)	.505			
Previous CRC, *n* (%)	8 (13.8)	14 (10.6)	.527			
BRAF-mut polyps, *n* (%)	0 (0)	2 (1.6)	.358			
KRAS-mut polyps, *n* (%)	17 (31.5)	25 (19.4)	**.076**	1.43	0.66–3.09	.369
CIMP+ polyps, *n* (%)	15 (25.9)	14 (10.6)	**.007**	2.31	1.00–5.36	**.049**
Polyp ≥ 10 mm, *n* (%)	36 (62.1)	73 (55.3)	.385			
≥3 polyps, *n* (%)	27 (46.6)	30 (22.7)	**.001**	2.58	1.31–5.08	**.006**
Villous component, *n* (%)	5 (8.6)	14 (10.6)	.674			
Advanced baseline adenomas, *n* (%)	36 (62.1)	73 (55.3)	.385			
**Patients with serrated polyps at baseline colonoscopy**						
	**Univariate analysis**			**Multivariate analysis**		
Variable	**Yes, *n* = 13 (30.2%)**	**No, *n* = 30 (69.8%)**	***p* value**	**OR**	**95% CI**	***p* value**
Age, mean (SD)	65.0 (13.7)	61.6 (12.2)	.482			
Male sex, *n* (%)	7 (53.8)	15 (50.0)	.817			
Previous smokers, *n* (%)	4 (36.4)	1 (4.3)	**.014**	15.9	0.50–501.8	.116
Previous CRC, *n* (%)	4 (30.8)	7 (23.3)	.608			
BRAF-mut polyps, *n* (%)	7 (53.8)	15 (50.0)	.817			
KRAS-mut polyps, *n* (%)	7 (53.8)	9 (30.0)	**.137**	3.2	0.33–29.7	.316
CIMP+ polyps, *n* (%)	6 (46.2)	3 (10.0)	**.007**	10.3	1.05–102.2	**.046**
Polyp ≥ 10 mm, *n* (%)	5 (38.5)	13 (43.3)	.766			
≥3 polyps, *n* (%)	10 (76.9)	12 (40.0)	**.026**	4.2	0.45–39.2	.207
Villous component, *n* (%)	0 (0)	1 (3.3)	.505			
Advanced baseline serrated lesions, *n* (%)	6 (46.2)	14 (46.7)	.975			

OR, odds ratio; CI, confidence interval; MACL, Metachronous Advanced Colorectal Lesions; SD, standard deviation; CRC, colorectal cancer.

Table S2. Time Until AML Diagnosis in Patients with CIMP+ Adenomatous and Serrated Lesions, adjusted for the Number of Colonoscopies Performed.

Time until AML development in patients with baseline adenomatous lesions						
		Univariate analysis		Multivariate analysis		
**Variable**		**Time in months, mean (SD)**	***p* value**	**HR**	**95% CI**	***p* value**
Sex	Men	46.5 (2.6)	.750			
	Women	44.0 (2.5)				
Previous smokers	Yes	30.2 (7.7)	.134	2.18	0.73–6.54	.163
	No	42.0 (2.3)				
Previous CRC	Yes	47.3 (5.6)	.801			
	No	43.7 (1.7)				
BRAF-mut polyps	Yes	--	.140			
	No	--				
KRAS-mut polyps, *n* (%)	Yes	45.2 (3.8)	.531			
	No	45.2 (2.0)				
CIMP+ polyps, *n* (%)	Yes	35.5 (3.0)	**.009**	2.78	1.19–6.47	**.018**
	No	49.1 (2.3)				
Polyp ≥ 10 mm, *n* (%)	Yes	45.2 (2.7)	.426			
	No	45.6 (2.5)				
≥3 polyps, *n* (%)	Yes	36.7 (3.6)	**<.001**	3.83	1.81–8.13	**<.001**
	No	48.4 (1.9)				
Villous component, *n* (%)	Yes	43.4 (6.0)	.749			
	No	46.3 (2.1)				
Advanced baseline adenomas, *n* (%)	Yes	45.2 (2.7)	.420			
	No	45.6 (2.5)				
**Time until AML development in patients with baseline serrated lesions**						
		**Univariate analysis**		**Multivariate analysis**		
**Variable**		**Time in months, mean (SD)**	***p* value**	**HR**	**95% CI**	***p* value**
Sex	Men	38.0 (4.0)	.241			
	Women	52.7 (4.7)				
Previous smokers	Yes	22.3 (8.3)	**.004**	2.25	0.41-12.52	.352
	No	48.0 (3.7)				
Previous CRC	Yes	46.0 (8.0)	.837			
	No	44.6 (3.5)				
BRAF-mut polyps	Yes	42.5 (5.1)	.500			
	No	50.7 (5.1)				
KRAS-mut polyps, *n* (%)	Yes	36.9 (5.8)	.052	4.53	1.13-19.04	**.039**
	No	54.1 (4.4)				
CIMP+ polyps, *n* (%)	Yes	33.1 (6.3)	**.042**	1.24	0.79-5.51	.775
	No	53.4 (4.3)				
Polyp ≥ 10 mm, *n* (%)	Yes	51.3 (5.6)	.559			
	No	43.0 (4.2)				
≥3 polyps, *n* (%)	Yes	36.0 (4.5)	**.020**	4.79	0.69-33.80	.117
	No	58.6 (4.2)				
Villous component, *n* (%)	Yes	--	.569			
	No	--				
Advanced baseline serrated lesions, *n* (%)	Yes	49.6 (5.6)	.814			
	No	43.8 (4.3)				

It was not possible to calculate the risk associated with BRAF-mut in adenomas, or with villous component in serrated lesions, since all events were censured. Multivariate analysis was adjusted for the number of colonoscopies (in adenomas: HR, 1.68; 95% CI, 1.1–2.5; p = .008; in serrated lesions: HR, 1.43; 95% CI, 0.7–2.9; p = .339). HR, hazard ratio; CI, confidence interval; MACL, Metachronous Advanced Colorectal Lesions; SD, standard deviation; CRC, colorectal cancer.

Table S3. Genes and Ligation Sites for Probes for CIMP Analysis.

Gene	Length (nt)	Ligation site
RUNX3	258	267nt before ex 2a reverse, 679 nt before ATG
	346	600-601,189 nt after ATG
	371	412-411 reverse, 0 nt before ATG
CACNA1G	218	573-572 reverse, 181 nt after ATG
	250	352-351 reverse, 40 nt before ATG
	218	102-103, 289 nt before ATG
IGF2	141	8 nt before ex 3, 824 nt before ATG
	172	318 nt after ex 4, 480 nt after ATG
	418	205-206, 611 nt before ATG
MLH1	130	47 nt before ex 1, 245 nt before ATG
	178	93 nt after ex 1, 209 nt after ATG
	355	460 nt before ex 1, 658 nt before ATG
	463	184 nt before ex 1 reverse, 382 nt before ATG
CRABP1	206	125-126, 21 nt after ATG
	265	255-256, 695 nt after ATG
	310	174 nt after ex 1, 245 nt after ATG
	318	108 nt after ex 2, 903 nt after ATG
SOSC1	155	102nt before ex 2, 152 nt before ATG
	238	416-417, 262 nt after ATG
	300	45nt before ex 1, 750 nt before ATG
	339	200 nt after ex 1, 400 nt before ATG
CDKN2A	184	102nt before ex 2a, 409 nt before ATG
	195	829 nt before ex 1, 990 nt before ATG
	232	434-433 reverse, 128 nt after ATG
	335	191-192, 31 nt after ATG
NEUROG1	166	581-580 reverse, 322 nt after ATG
	201	157 nt before ex 1, 417 nt before ATG
	212	168-167 reverse, 92 nt before ATG
	283	258-259, 2 nt before ATG
	364	38 nt before ex 1, 297 nt before ATG
	391	227 nt before ATG

nt-nucleotides; ex-exon
